# Measuring health literacy: A systematic review and bibliometric analysis of instruments from 1993 to 2021

**DOI:** 10.1371/journal.pone.0271524

**Published:** 2022-07-15

**Authors:** Mahmoud Tavousi, Samira Mohammadi, Jila Sadighi, Fatemeh Zarei, Ramin Mozafari Kermani, Rahele Rostami, Ali Montazeri

**Affiliations:** 1 Health Metrics Research Center, ACECR, Iranian Institute for Health Sciences Research, Tehran, Iran; 2 Faculty of Medical Sciences, Department of Health Education, Tarbiat Modares University, Tehran, Iran; 3 Faculty of Humanity Sciences, University of Science and Culture, Tehran, Iran; Universidad Nacional de Caaguazu, PARAGUAY

## Abstract

**Background:**

It has been about 30 years since the first health literacy instrument was developed. This study aimed to review all existing instruments to summarize the current knowledge on the development of existing measurement instruments and their possible translation and validation in other languages different from the original languages.

**Methods:**

The review was conducted using PubMed, Web of Science, Scopus, and Google Scholar on all published papers on health literacy instrument development and psychometric properties in English biomedical journals from 1993 to the end of 2021.

**Results:**

The findings were summarized and synthesized on several headings, including general instruments, condition specific health literacy instruments (disease & content), population- specific instruments, and electronic health. Overall, 4848 citations were retrieved. After removing duplicates (n = 2336) and non-related papers (n = 2175), 361 studies (162 papers introducing an instrument and 199 papers reporting translation and psychometric properties of an original instrument) were selected for the final review. The original instruments included 39 general health literacy instruments, 90 condition specific (disease or content) health literacy instruments, 22 population- specific instruments, and 11 electronic health literacy instruments. Almost all papers reported reliability and validity, and the findings indicated that most existing health literacy instruments benefit from some relatively good psychometric properties.

**Conclusion:**

This review highlighted that there were more than enough instruments for measuring health literacy. In addition, we found that a number of instruments did not report psychometric properties sufficiently. However, evidence suggest that well developed instruments and those reported adequate measures of validation could be helpful if appropriately selected based on objectives of a given study. Perhaps an authorized institution such as World Health Organization should take responsibility and provide a clear guideline for measuring health literacy as appropriate.

## Introduction

The term ‘health literacy’ was first used in 1974 in a paper entitled ‘health education as a social policy’ [1]. Since then, health literacy appeared more frequently in the biomedical literature and believed that it goes beyond the ability to read, write, and understand the meanings of words and numbers in health care settings [2]. The World Health Organization (WHO) defined health literacy as: ‘cognitive and social skills that determine the motivation and ability of individuals to access understand and use the information to promote and maintain optimal health’ [3]. Later the WHO regional office for Europe defined health literacy as: ‘Health literacy is linked to literacy and entails people’s knowledge, motivation and competences to access, understand, appraise and apply health information in order to make judgments and take decisions in every- day life concerning health care, disease prevention and health promotion to maintain or improve quality of life during the life course’ [4].

Health literacy is believed to have a vital impact on public health through access to and use of health services [5, 6]. Low health literacy is associated with poor health status [6, 7], frequent use of health services, longer hospital length of stay [5, 6], and high mortality [7, 8]. In addition, some studies have linked low health literacy to unhealthy behaviors, such as smoking [4, 9–12], low physical activity [10–12], and low use of preventive services [4, 7, 10]. Essentially, health literacy plays a role in improving health outcomes both at the individual level (reducing health inequalities) and at the societal level (continuous development of health policies) [13].

Therefore, measuring health literacy is fundamental and needs appropriate measures. Among health literacy instruments, the Rapid Assessment of Adult Literacy in Medicine (REALM) [14], the Test of Functional Health Literacy (TOFHLA) [15], and the Newest Vital Sign (NVS) [16] have a long history of application. These instruments have been criticized for a number of reasons, including evaluation of only a few areas of health literacy, inadequacy for use in interventional studies, or lack of development with a health promotion perspective. In addition, most of these scales were developed and used in clinical settings [17].

In a review of the literature from 1999 to 2013, 51 instruments were identified. Of these, 26 were general health literacy instruments, 15 were condition specific (disease or content), and 10 were health literacy instruments in a specific population [18]. In a review by O`Neil et al. on self-administered health literacy instruments, 35 measures were reported (27 original; 8 derivative instruments) [19]. Nguyen et al., in their study, stated that there are more than 100 health literacy instruments, but only a small number of them have been developed using modern guidelines [20]. In addition, there were further review papers with limited focus covering either general measures or papers that reviewed condition and population- specific health literacy measures. A chronological list of selected review papers is provided in Table 1 [20–38]. However, none of the previous reviews assess instruments comprehensively. Thus, to provide insight into the literature, we performed a bibliometric analysis from the start to the end of 2021 to comprehensively review all existing instruments. We thought this might help synthesize evidence and provide a platform for investigators with similar interests to easily select, apply, or appraise an instrument when needed.

**Table 1:** Review papers on health literacy instruments.

Author [ref.]	Year	Number of instruments reviewed	focus
Machado et al. [21]	2014	4	Health literacy in elderly hypertensive patients
Dickson-Swift et al. [22]	2014	32	Oral health literacy
O’Connor et al. [23]	2014	13	Mental health literacy
Parthasarathy et al. [24]	2014	13	Oral health literacy
Perry [25]	2014	5	Health literacy in adolescents
Wei et al. [26]	2015	Validated measures: knowledge (14), stigma (65), help-seeking related (10)	Mental health literacy (knowledge, stigma, help-seeking related)
Duell et al. [27]	2015	43	Health literacy in a clinical setting
Stonbraker et al. [28]	2015	19	Health literacy among Spanish speakers in clinical or research settings
Nguyen et al. [20]	2015	Instruments (109): General HL (58), specific content/context (51)	Health literacy measures for ethnic minority populations
Wei et al. [29]	2017	12	Mental health literacy tools measuring help-seeking
Lee et al. [30]	2017	13	Health literacy for people with diabetes
Shum et al. [31]	2018	Asthma (40), COPD (22), Asthma/COPD (3)	Airway diseases and health literacy measurement tools
Guo et al. [32]	2018	29	Children and adolescents
Wei et al. [33]	2018	101	Mental health literacy measurement tools (the stigma of mental illness)
Okan et al. [34]	2018	15	Health literacy instruments used in children and adolescents
Estrella et al. [35]	2020	17	Health literacy among US African Americans and Hispanics/Latinos with type 2 diabetes
Slatyer et al. [36]	2020	3	Self-reported instruments to assess health literacy in older adults
Ghaffari et al. [37]	2020	21	Oral and dental health literacy
Mafruhah et al. [38]	2021	48	Health literacy for medication use

## Materials and methods

### Search engine and time period

The electronic databases searched included PubMed, Scopus, Web of Science, and Google Scholar. The aim was to review all full publications in biomedical journals between 1993 and 2021. The search was updated twice: once in January 2022 and once in early February 2022. The year 1993 was chosen since the first standard instrument was reported in 1993.

### Search strategy

The search strategy was limited to health literacy instruments whose psychometric information was accurately and transparently presented. Papers were retrieved using different combinations of keywords and MeSH terms including; ‘Health literacy’, ‘eHealth literacy’, ‘e-Health literacy’, ‘e Health literacy’, ‘electronic Health literacy’, ‘Tool’, ‘Instrument’, ‘Scale’, ‘Questionnaire’, ‘Measure’ and ‘Inventory’ in the title and abstract of papers.

All potentially relevant publications were extracted and reviewed independently by two authors (SM and FZ). Discrepancies between authors were resolved by consensus with the first investigator (MT). Then, qualified studies were obtained for full‐text screening. The three authors extracted the data in order to identify eligible studies. After the final evaluation, the required data were extracted and recorded.

### Ethics statement

The Iranian Academic Center for Education, Culture, and Research (ACECR) approved the study (Code of Ethics approved: IR.ACECR.IBCRC.REC.1397.014).

### Selection criteria

This study included all original papers reporting psychometric properties of health literacy (and e-health literacy) instruments published in English. Papers only published in journals remained in the study, and books and pamphlets, dissertations, papers presented at conferences, etc., were excluded. All publications were screened using the PRISMA guideline [39].

### Quality assessment

The quality of papers was evaluated using the Consensus-based Standards for the selection of the health status Measurement Instrument (COSMIN) checklist. The COSMIN initiative aims to improve the selection of health measurement instruments [40]. For the purpose of this review reporting, six criteria (with at least eight items) were considered sufficient, and for each reported item, a score of 1 was assigned, giving a total score of 8. The criteria were reporting: internal consistency, stability (interclass correlation), face/content validity, structural validity (exploratory and confirmatory factor analyses), criterion validity, hypotheses testing (convergent or divergent validity, discriminant or known groups comparison). Then, the quality of psychometric reporting of each measure was categorized as: poor (< 2), fair (2, 3), good (4, 5), and excellent (≥ 6).

### Data synthesis

The data for each paper were extracted and summarized. The summary then was tabulated by a topic. The following information was provided: author(s)’ name, year of publication, validity, and reliability, and type of instruments, including: ‘general health literacy instruments’, ‘condition (disease or content) specific instruments’, instruments that were developed for ‘specific populations’ [18], and e-Health Literacy instruments.

## Results

### Descriptive findings

**Fig 1:**
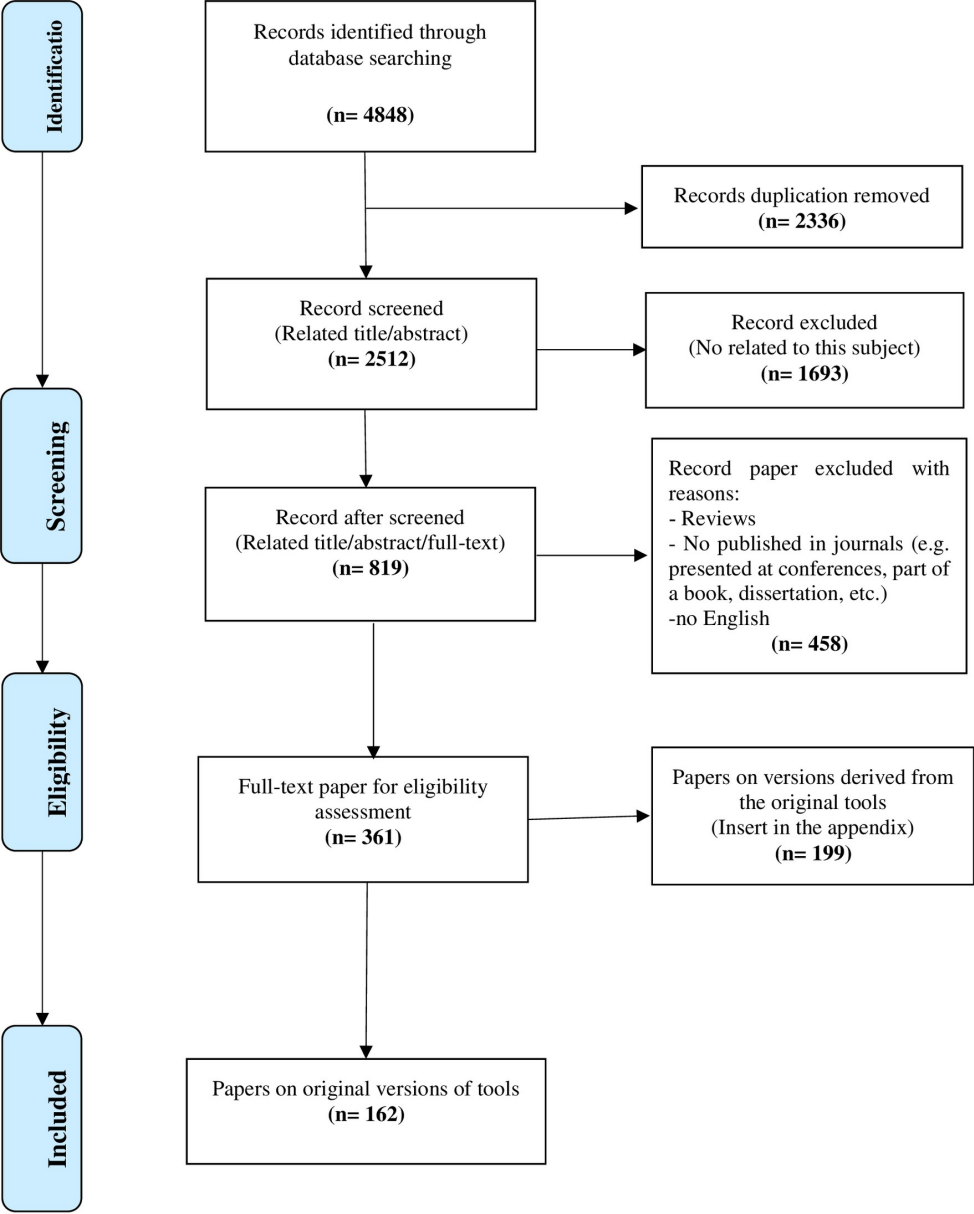
Flow diagram of the study selection process.

The study flowchart is presented in Fig 1. Overall, 4848 papers were identified. After removing duplicates (n = 2336) and irrelevant documents (n = 2175), 361 papers were included in the final review. Of these, 162 papers introduced an instrument, and 199 papers reported translation and psychometric properties for an original measure. Indeed, the original instruments are briefly described in four categories in the following sections.

### General health literacy instruments

There were 39 instruments for measuring general health literacy. Historically among the general instruments, the most frequently used instruments were the REALM [14], the TOFHLA [15], and the NVS [16]. However, recently two well-developed instruments were introduced: The Health Literacy Questionnaire (HLQ) [55] and the Health Literacy Survey Questionnaire (HLS-EU-Q) [56]. The HLS-EU-Q and its newer versions [61, 69] have been widely used in European and Asian settings. Overall proper psychometric properties were reported for measures in this category. A summary of findings is presented in Table 2.

**Table 2:** General health literacy instruments (1993–2021).

Author [ref.]	Year	Name (abbreviation)	Country/sample	Items	Validity		Reliability	
					Face/Content	Construct	Internal consistency	External/Relative
Davis et al. [14]	1993	Rapid estimate of adult literacy in medicine (REALM)	American public health and primary care settings	66	-	Concurrent	Cronbach α = 0.86	Test-retest = 0.99
Parker et al. [15]	1995	Test of Functional Health Literacy in Adults (TOFHLA)	American adults patients	57	✓	Concurrent	Cronbach α = 0.98	-
Baker et al. [41]	1999	Short form of the Test of Functional Health Literacy in Adults (S-TOFHLA)	American English speaking patients	40	-	Concurrent	Cronbach α = 0.97	-
Weiss et al. [16]	2005	Newest Vital Sign (NVS)	American adults	6	-	Concurrent	Cronbach α = 0.78	-
Lee et al. [42]	2006	Short Assessment of Health Literacy for Spanish-speaking Adults (SAHLSA-50)	American Spanish-speaking adults	50	-	Convergent; Predictive; CFA	Cronbach α = 0.92	Test-rest = 0.86
Morris et al. [43]	2006	Single Item Literacy Screener (SILS)	American adults with diabetes	1	-	Criterion	-	-
Zikmund-Fisher et al. [44]	2007	Subjective Numeracy Scale (SNS)	American general population	8	-	Predictive	-	-
Ishikawa et al. [45]	2008	Functional, Communicative, and Critical Health Literacy (FCCHL)	Japanese diabetic patients	14	-	Discriminant; EFA	Cronbach α = 0.65–0.84	-
Chew et al. [46]	2008	3 health literacy screening questions	American adult patients	3	-	Criterion	-	-
Pleasant et al. [47]	2008	Public health literacy knowledge scale	Mexican & Chinese & Ghanaian& Indian participants	16	✓	Discriminate	Cronbach α = 0.79	-
Rawson et al. [48]	2009	Medical Term Recognition Test (METER)	American adult patients	40	-	Predictive	Cronbach α = 0.93	-
Zhang et al. [49]	2009	Functional Health Literacy Tests (FHLTs)	Singapore: general public and rheumatic patients	21	-	Divergent (Discriminant); Convergent	Cronbach α = 0.72, 0.68	Test-retest = 0.56; ICC = 0.95
McCormack et al. [50]	2010	Health literacy skills instrument	American population	25	✓	CFA; Concurrent	Cronbach α = 0.86; Item-total correlation = 0.27–0.59	-
Yu Ko et al. [51]	2012	Health Literacy Test for Singapore (HLTS)	Singapore adults	25	✓	Convergent; Predictive	Cronbach α = 0.87	-
Begoray et al. [52]	2012	Self-reported health literacy scale	Canadian adults	9	-	Criterion	Cronbach α = 0.83	-
Kaphingst et al. [53]	2012	Health literacy INDEX: health literacy demands of health information materials	American adults	63	-	Concurrent	-	kappa value = 0.6–0.64
Helitzer et al. [54]	2012	The TALKDOC health literacy measurement tool	New Mexico female adults	80	✓	Convergent	-	-
Osborne et al. [55]	2013	Health Literacy Questionnaire (HLQ)	Australian general population	44	✓	CFA; Discriminant	Cronbach α = 0.86–0.90	-
Sorensen et al. [56]	2013	Health Literacy Survey Questionnaire (HLS-EU-Q-47)	English/Bulgarian/Dutch/German/Greek/Polish/Spanish/Irish/Austrian adults	47	✓	EFA	Cronbach α = 0.51–0.91	-
Suka et al. [57]	2013	14-item Health Literacy Scale (HLS-14)	Japanese adults	14	-	EFA; CFA	Cronbach α = 0.76–0.85	-
Farin et al. [58]	2013	Health Education Literacy of Patients (HELP questionnaire)	German patient adults	18	✓	EFA; CFA; IRT	Cronbach α = 0.88–0.95	-
Jordan et al. [59]	2013	The Health Literacy Management Scale (HeLMS)	Australian adults	29	✓	EFA; CFA	Cronbach α> 0.82	ICC> 0.90
Sand-Jecklin [60]	2014	Brief Health Literacy Screen (BHLS)	American adult patients	5	-	EFA; Concurrent	Cronbach α = 0.79	-
Pelikan et al. [61]*	2014	Short versions of the European Health Literacy Survey Questionnaire (HLS-EU-Q16, Q6)	English/Bulgarian/Dutch/German/Greek/Polish/Spanish/Irish/Austrian adults	16 & 6	✓	CFA; Concurrent	Cronbach α = 0.80 for Q6	-
Kang et al. [62]	2014	Korean Health Literacy Instrument (KHLI)	Korean adults	18	✓	EFA; CFA	Cronbach α = 0.82	Test-retest = 0.89
Nakagami et al. [63]	2014	Japanese Functional Health Literacy Test (JFHLT)	Japanese adults	16	✓	Convergent; Concurrent	Cronbach α = 0.81	-
Chau et al. [64]	2015	Chinese Health Literacy Scale for Low Salt Consumption-Hong Kong population (CHLSalt-HK)	Hong Kong older adults	49	✓	Discriminant; EFA; CFA; Concurrent t; Predictive	Cronbach α = 0.79	Test-retest = 0.84; ICC = 0.7
Haghdoost et al. [65]	2015	Iranian Health Literacy Questionnaire (IHLQ)	Iranian adults	36	✓	EFA	Cronbach α = 0.71–0.96	Test-retest [ICC] = 0.73 to 0.86
Zotti et al. [66]	2017	Single question on Self-rated Reading Ability (SrRA)	Italian adult cancer patients	1	✓	Convergent; Discriminant	-	-
Tsubakita [67]	2017	Functional Health Literacy Scale for Young Adults (funHLS-YA)	Japanese Young Adults	19	-	Criterion; EFA	Cronbach α = 0.75	-
Kim [68]	2017	short version of the Korean Functional Health Literacy Test (S‐KHLT)	Korean nursing students and older adults	8	-	Convergent;	KR‐20 = .84	-
Finbraten et al. [69]	2018	Short version of the European Health Literacy Survey Questionnaire (HLS-Q12)	Norwegian adults	12	-	Rasch model; CFA; Convergent	Person separation Reliability = 0.75–0.82	-
Pleasant et al. [70]	2018	Calgary charter on health literacy scale	American general population	5	-	Discriminant	Cronbach α = 0.80	-
Duong et al. [71]	2019	European Health Literacy Survey questionnaire (HLS-SF12)	Indonesian/Kazakh/Russian/Malay/Myanmar/Burmese/Mandarin/Vietnamese adults	12	-	Convergent; CFA	Cronbach α = 0.85	-
Mc Clintock et al. [72]	2020	Eight health literacy questions based on the national academy of medicine	Sub-Saharan Africa countries adults	8	✓	Discriminant; EFA	Cronbach α = 0.72	-
Leung et al. [73]	2020	Rapid Estimate of Inadequate Health Literacy for older adults (REIHL)	Hong Kong patients with chronic illnesses	12	-	Concurrent	Sensitivity and specificity (by ROC curve analysis)	-
Shannon et al. [74]	2020	Health Communication Questionnaire (HCQ)	Australian mining industry workers	14	✓	-	-	Test-retest = 0.72
Tavousi et al. [75]	2020	Health Literacy Instrument for Adults (HELIA)	Iranian adults	33	✓	EFA	Cronbach α = 0.72–0.89	-
Park et al. [76]	2021	Korean Health Literacy Instrument	Late School-Aged Children	16	✓	EFA, CFA, Criterion	KR-20 = 0.85, 0.88, 0.82 & item-total correlations = 0.31–0.69	-

*Unpublished (conference).

### Condition (disease or content) specific instruments

There were 90 condition specific (disease & content) instruments. Measuring health literacy for chronic non-communicable diseases, especially diabetes mellitus, has been considered more frequently. At least nine instruments assess health literacy in diabetes. Infectious diseases (such as HIV, HPV, tuberculosis, cholera, and infectious disease-specific) were the second topic of interest in developing health literacy measures. These instruments have also been well-reviewed and validated in relevant studies in terms of validity and reliability (Table 3).

**Table 3:** Disease specific health literacy instruments (1993–2021).

Author [ref.]	year	Name (abbreviation)	Country/sample	Disease	Items	Validity		Reliability	
						Face/Content	Construct	Internal consistency	External
Huizinga et al. [77]	2008	Diabetes Numeracy Test (DNT43, 15)	English patients	Type 2 diabetes	43 & 15	✓	Discriminant; Convergent; EFA	KR-20 = 0.95 & 0.90	-
Kim et al. [78]	2012	High Blood Pressure- focused Health Literacy Scale (HBP-HLS)	Korean American elder (aged 60 or older)	High blood pressure	30	✓	Convergent; Discriminant	KR-20 = 0.98	-
Leung et al. [79]	2013	Chinese Health Literacy Scale for Diabetes (CHLSD)	Chinese patients elder (aged 65 or older)	Type 2 diabetes	34	✓	Discriminant; CFA	Cronbach α = 0.65–0.88	Test-retest = 0.89
Leung et al. [80]	2013	Chinese Health Literacy scale for Chronic Care (CHLCC)	Chinese patients elder (aged 65 or older)	Chronic illnesses (hypertension, diabetes mellitus, chronic obstructive pulmonary disease, or arthritis)	24	✓	Discriminant	Cronbach α = 0.91	Test-retest (ICC) = 0.77
Ownby et al. [81]	2013	Brief computer-administered HIV-related Health Literacy Scale (HIV-HL)	American physicians	Treated for HIV infection	19	-	Convergent; Concurrent; EFA	Cronbach α = 069	-
Sun et al. [82]	2013	Skills-based instrument on health literacy regarding respiratory infectious diseases	Chinese patients	Respiratory infectious diseases	30	-	EFA; CFA	Cronbach α = 0.86; Item-total relation = 0.86	-
Han et al. [83]	2014	Assessment of Health Literacy in Cancer screening (AHL-C)	Korean American immigrant women	Breast and cervical cancer screening	52	✓	Convergent; Concurrent; Discriminant	Cronbach α = 0.96; Item-total correlations = 0.18–0.86	-
Dumenci et al. [84]	2014	Cancer Health Literacy Test (CHLT-30) & (CHLT-6)	American English speaking adults	Cancer	30 & 6	✓	CFA; Discriminant	Cronbach α = 0.88	Test-retest = 0.90 (for CHLT-30)
Londono et al. [85]	2014	Tool for asthma patients in the Italian-speaking	Italian-speaking patient’s region of Switzerland	Asthma	19	✓	-	-	ICC = 0.97
Shih et al. [86]	2016	Health literacy questionnaire for Taiwanese hemodialysis patients	Taiwanese adult patients	Hemodialysis	26	✓	CFA	Cronbach α = 0.81	-
Matsuoka et al. [87]	2016	Heart Failure-specific Health Literacy scale (HF-specific HL)	Japanese patients adults with HF	Heart failure	12	✓	EFA; Discriminant	Cronbach α = 0.71	Test-retest (ICC) = 0.88–0.89
Tian et al. [88]	2016	Infectious Disease-Specific Health Literacy (IDSHL)	Chinese population adults households	Infectious disease-specific	22	✓	EFA; Discriminant	Cronbach α = 0.75–0.81; item-total correlation (<0.30)	-
Mafutha et al. [89]	2017	Hypertension Health Literacy Assessment Tool (HHLAT)	South African adult patients	Hypertension	11	✓	Concurrent	-	-
Tique et al. [90]	2017	HIV Literacy Test (HIV-LT)	Portuguese speaking patients	HIV infection	16 & 10	✓	EFA; Convergent	KR-20 = 0.87	-
Chou et al. [91]	2017	Cancer Health Literacy Scale (C-HLS)	Chinese adults patients	Newly diagnosed cancer patients	33	✓	CFA; Criterion	Spearman–Brown split-half coefficient = 0.74; KR-20 = 0.82	-
Yang et al. [92]	2018	Infectious disease-specific health literacy (IDSHL)	General population of Tibet	Infectious disease fever, diarrhea, rash, jaundice or conjunctivitis)	25	-	CFA; Known-groups	Cronbach α = 0.70; split-half coefficient = 0.62	-
Lee et al. [93]	2018	Comprehensive Diabetes Health Literacy Scale (DHLS)	Korean adults	Diabetes	14	✓	Criterion; Convergent; EFA; CFA	Cronbach α = 0.91	Test-retest (ICC) = 0.89
Khazaei et al. [94]	2018	Heart Health Literacy Scale (HHLS)	Iranian adults	Heart health literacy	26	✓	EFA; CFA	Cronbach α = 0.88	Test-retest = 0.81
Dehghani et al. [95]	2018	Multidimensional Health Literacy Questionnaire for multiple sclerosis patients (MSHLQ)	Iranian patients	Multiple sclerosis	22	✓	EFA; Known-groups	Cronbach α = 0.94	ICC = 0.96
Yeh et al. [96]	2018	Diabetes-specific health literacy	Mandarin/Taiwanese-speaking patients	Type 2 diabetes	11	✓	CFA	KR-20 = 0.84	-
Kanga et al. [97]	2018	Korean Health Literacy Scale for Diabetes Mellitus (KHLS-DM)	Korean diabetic patients	Type 2 diabetes	58	✓	Rasch analysis; EFA; Criterion; CFA	Cronbach α = 0.83	Test-retest = 0.80
Tutu et al. [98]	2019	Household cholera-focused health literacy scale	American households urban poor	Household cholera-focused	13	✓	EFA	Cronbach α = 0.76	-
Cardoso et al. [99]	2019	Alfabetizacao em Saude Relacionada a Adesao Medicamentosa entre Diabeticos (ASAM-D)	Brazilian diabetic patients adults	Type 2 diabetes	18	✓	-	Cronbach α = 0.77	Kappa coefficient = 0.31–1
De Sousa et al. [100]	2019	Instrument of the Health Literacy regarding Diabetic Foot (HLDF)	Brazilian diabetic patients adults	Diabetic foot	18	✓	Concurrent	Cronbach α = 0.73	ICC = 0.79; Kappa< 0.60
Li et al. [101]	2019	Chinese Health Literacy Scale for Tuberculosis (CHLS-TB)	Chinese patients	Tuberculosis	31	✓	EFA; CFA; Discriminant	Cronbach α = 0.0.82, split-half reliability = 0.78	Test-retest = 0.95
Wu et al. [102]	2020	Brief tool to measure melanoma-related health literacy and attitude	Chinese adolescents	Melanoma	13	✓	CFA	Spear-Brown split-half = no reported	Kappa coefficient> 0.7
Martins et al. [103]	2020	Oral Health Literacy among Diabetics (OHL-D)	Brazilian adults	Type 2 diabetes	30	✓	-	-	Kappa coefficient> 1
Echeverri et al. [104]	2020	Multidimensional Cancer Literacy Questionnaire (MCLQ)	American diverse populations	Cancer	82	-	Content; EFA; CFA; Discriminant	Cronbach α = 0.89	-
Huang et al. [105]	2020	Health Literacy battery for three phases of Stroke (HL-3S)	Taiwanese adults patients	Stroke survivors	30	-	Rasch analysis	Rasch reliability coefficients = 0.86 and 0.87	-
Rajabi et al. [106]	2020	Health literacy questionnaire on the most important domains of Non Communicable Diseases (NCDs)	Iranian patient	Cardiovascular diseases, diabetes, and cancer	27	✓	EFA	Cronbach α = 0.93	-
Wei et al. [107]	2021	health literacy specific to Chronic Kidney Disease (CKD)	Taiwanese patients	Chronic kidney disease (CKD)	17	✓	CFA	KR-20 = 0.68	-
Chen et al. [108]	2021	Health Literacy Assessment Instrument	Chinese patients	Chronic Pain	31	✓	EFA; CFA	Cronbach α = 0.93–0.97; split-half reliability = 0.91	Test-retest = 0.93
Savci et al. [109]	2021	Health Literacy Scale for Protection Against COVID-19	Turkish Adults (15–30)	COVID-19	20	✓	EFA; CFA; Criterion	Cronbach α = 0.97; item-total correlation = 0.68–0.94	-
Hiltrop et al. [110]	2021	COVID-19 related Health Literacy in Healthcare Professionals (HL-COV-HP)	Healthcare professionals	COVID-19	12	-	EFA; CFA; Convergent	Cronbach α = 0.87	-

Among the instruments with special content, the most frequently used were oral/dental health literacy and mental health literacy. The parental and maternal, insurance, occupational, complementary, and alternative medicine, the responsiveness of primary care practices, weight-specific childhood, overweight, social determinants of health, and non-specific neck pain health food, were other specific content measures (Table 4).

**Table 4:** Content specific health literacy instruments (1993–2021).

Author [ref.]	year	Name (abbreviation)	Country/sample	Condition	Items	Validity		Reliability	
						Face/Content	Construct	Internal consistency	External
Cormier et al. [111]*	2006	Health Literacy Knowledge and Experience Survey (HL-KES)	American nursing students	Knowledge and experience	38	✓	EFA	Cronbachα = 0.79, 0.76	-
Sabbahi et al. [112]	2009	Oral Health Literacy Instrument (OHLI)	Canadian adults	Oral health literacy	57	✓	Convergent; Discriminant; Concurrent	Cronbach α = 0.89	ICC = 0.88
Kumar et al. [113]	2010	Health Literacy, numeracy and the Parental Health Literacy Activities Test (PHLAT)	American caregivers of infants	Parental health literacy	10 & 20	✓	Discriminant	KR-20 = 0.76	-
Macek et al. [114]	2010	Comprehensive oral health knowledge	American low-income adults	Oral health literacy	4	✓	Criterion	Cronbach α = 0.74	-
Devi et al. [115]	2011	Questionnaire to assess oral health literacy among college students in Bangalore city	Indian college students	Oral health literacy	14	-	Convergent; Predictive	Cronbachα = 0.40	Test-retest = 0.69
Mojoyinola [116]	2011	Maternal Health Literacy and Pregnancy Outcome Questionnaire (MHLAPQ)	All pregnant women patients	Maternal health literacy	33	-	-	Cronbach α = 0.81	-
Loureiro et al. [117]	2012	Questionario de Avaliacao da Literacia em Saude Mental (QuALiSMental)	Portuguese adolescents and young people	Mental health literacy	46	-	EFA	Cronbach α = 0.60–0.82	-
Wong et al. [118]	2013	Hong Kong Oral Health Literacy Assessment Task for Pediatric dentistry (HKOHLAT-P)	Speak Chinese child/parent dyads in Hong Kong	Oral health literacy	2	✓	Convergent; Predictive t; Concurrent	Cronbach α = 0.86, 0.73	Test-retest (ICC) = 0.63
Dahlke et al. [119]	2014	Mini Mental Status Exam (MMSE)	American English speaking older adults	Mental health literacy	5	✓	Convergent; Criterion (Predictive)	-	-
Jones et al. [120]	2014	Health Literacy in Dentistry scale (HeLD-29)	Indigenous Australians adults	Oral health literacy	29	✓	Convergent; Predictive; Discriminant; EFA	Cronbach α = 0.91	ICC = 0.65
Naghibi Sistani et al. [121]	2014	Oral Health Literacy for Adults Questionnaire (OHL-AQ)	Iranian adults	Oral health literacy	17	✓	Discriminant	Cronbach α = 0.72	Test-retest (ICC) = 0.84
Paez et al. [122]	2014	Health Insurance Literacy Measure (HILM)	American adult	Health insurance literacy	42	-	EFA; CFA; Convergent	Cronbach α> 0.9	-
Shreffler-Grant et al. [123]	2014	Montana State University (MSU) CAM health literacy scale	American older adults living in rural	Complementary and alternative medicine	21	✓	Convergent; EFA	Cronbach α = 0.75	-
Villanueva Vilchis et al. [124]	2015	Spanish Oral Health Literacy Scale (SOHLS)	Mexican adult	Oral health literacy	29	✓	Convergent	Cronbach α = 0.74	Test-retest (ICC) = 0.76
O’Connor et al. [125]	2015	Mental Health Literacy Scale (MHLS)	Australian residents	Mental health literacy	35	✓	EFA; Concurrent; Discriminant	Cronbach α = 0.87	Test-retest = 0.79
Altin et al. [126]	2015	Health Literacy responsiveness of Primary Care practices (HLPC)	German general population	Primary care practices	4	-	EFA; CFA; Concurrent	Cronbach α = 0.86	-
Curtis et al. [127]	2015	Comprehensive Health Activities Scale (CHAS)	American participants	Comprehensive health activities	45	-	Predictive; Convergent; CFA	Cronbach α = 0.92	-
Guttersrud et al. [128]	2015	Maternal Health Literacy (MaHeLi) scale	Uganda adolescents patients	Maternal health literacy	12	-	Rasch models	Cronbach α = 0.92; Person Separation Index (PSI) = 0.82–0.90	-
Stein et al. [129]	2015	Adult Health Literacy Instrument for Dentistry (AHLID)	Norwegian adults older	Oral health literacy	-	✓	Predictive	Cronbach α (= 0.98)	Test-retest = 0.81
Intarakamhang et al. [130]	2016	Alcohol, Baccy, Coping, Diet, and Exercise Health Literacy scale (ABCDE-HL)	Thai adults	ABCDE	64	✓	EFA; CFA	Cronbach α = 0.61–0.91	-
Kapoor et al. [131]	2016	Determination of Functional Literacy in Dentistry (DFLD)	Indian patients	Oral health literacy	30words/30 items	✓	Convergent; Predictive	Cronbach α = 0.84	Test-retest = 0.69
Jung et al. [132]	2016	Multicomponent mental health literacy measure	American local public housing authority	Mental health literacy	26	✓	Groups known; EFA; CFA; Convergent	Cronbach α = 0.76–0.84; KR-20 = 0.83	-
Campos et al. [133]	2016	Mental Health Literacy questionnaire (MHLq)	Portuguese young people	Mental health literacy	33	✓	EFA	Cronbach α = 0.84	Test-retest (ICC) = 0.88
Squires et al. [134]	2017	Health literacy promotion practices assessment instrument	American health care provider	Health promotion practices	38	✓	EFA	Cronbach α = 0.95	-
Bjornsen et al. [135]	2017	Mental Health-Promoting Knowledge (MHPK-10)	Norwegian adolescents	Mental health literacy	10	✓	Groups known; EFA; CFA	Cronbach α = 0.87	Test-retest = 0.70
Moll et al. [136]	2017	Mental Health Literacy tool for the Workplace (MHL- W)	Canadian healthcare workers	Mental health literacy	16	-	Discriminant; Convergent; EFA	Cronbach α = 0.94	-
Intarakamhang et al. [137]	2017	HL scale for Thai childhood overweight	Thai school students	Childhood overweight	Childhood overweight	55	-	EFA; CFA	Cronbach α = 0.70; KR-20 = 0.76; Item-total correlation coefficient = 0.2–0.8	-
Childhood overweight
Matsumoto et al. [138]	2017	Health Literacy of Social Determinants of Health Questionnaire (HL-SDHQ)	Japanese adults	Social determinants of health	33	✓	CFA	Cronbach α = 0.92	-
Tsai et al. [139]	2018	Weight-Specific Health Literacy Instrument (WSHLI)	Taiwanese adults	Weight-Specific	✓	-	Convergent; Predictive; EFA; CFA	Cronbach α = 0.80 & 0.81; split-half coefficient = 0.78 & 0.81	-
Lichtveld et al. [140]	2019	Environmental Health Literacy (EHL)	American public health students	Environmental health literacy	42	✓	EFA; CFA	Cronbach α = 0.63–0.70	-
Areerak et al. [141]	2019	Neck pain-specific Health Behavior in Office Workers (NHBOW)	Thai office workers	Non-specific neck pain	6	✓	EFA; CFA; Discriminative	Cronbach α = 0.64, 0.53	Test-retest (ICC) = 0.75
Zhang et al. [142]	2019	Chinese Parental Health Literacy Questionnaire (CPHLQ)	Chinese caregivers of children (0–3 years)	Parental health literacy	39	✓	CFA	Cronbach α = 0.89; Spilt-half (Spearman-Brown coefficient) = 0.92	Test-retest = 0.82
Irvin et al. [143]	2019	Water Environmental Literacy Level Scale (WELLS)	Thai adults office workers	Water environmental literacy	6	✓	Criterion; Discriminative	Cronbach α = 0.51	-
Wei et al. [144]	2019	Mental Health Literacy tool for Educators (MHL-ED)	Canadian educators	Mental health literacy	29	✓	EFA; Groups known;	Cronbach α = 0.85	-
Ayre et al. [145]	2020	Parenting Plus Skills Index (PPSI)	Australian parents	Parenting health literacy	13	✓	CFA; Criterion	Cronbach α = 0.70	-
Intarakamhang et al. [146]	2020	Environmental Health Literacy (EHL)	Thai village health volunteers	Environmental health literacy	25	✓	CFA	Cronbach α = 0.91–0.93	-
Suthakorn et al. [147]	2020	Thai Occupational Health Literacy Scale- Informal Workers (TOHLS-IF)	Thai informal workers	Occupational health literacy	38	✓	EFA; CFA	Cronbach α = 0.98	-
Lin et al. [148]	2020	Chinese Medication Literacy Measurement (ChMLM-13 &17)	Mandarin or Taiwanese adults	Medication-related health literacy	13 & 17	✓	EFA; Convergent; Discriminant	Cronbach α = 0.83, 0.78	-
Taheri et al. [149]	2020	Maternal Health Literacy Inventory in Pregnancy (MHELIP)	Iranian pregnant women	Maternal health literacy	48	✓	EFA	Cronbach α = 0.94	ICC = 0.96
Tabacchi et al. [150]	2020	Food Literacy Assessment Tool (FLAT)	Italian children	Food literacy	16	✓	Discriminant; CFA	Cronbach α = 0.73 to 0.76	-
Zenas et al. [151]	2020	Danish Mental Health Literacy Adolescents questionnaire (MeHLA)	Danish adolescents	Mental health literacy	Not indicated-	✓	EFA; CFA	Cronbach α = 0.82	-
Taoufik et al. [152]	2020	Greek Oral Health Literacy measurement instrument (GROHL-20)	Greece adult patients	Oral health literacy	20	✓	Convergent	Cronbach α = 0.80	Test-retest (ICC) = 0.95
Chao et al. [153]	2020	Mental Health Literacy Scale for Healthcare Students (MHLS-HS)	Taiwanese health care students	Mental health literacy	26	✓	EFA; CFA; Convergent; Discriminant; Known groups	Cronbach α = 0.70–0.87	-
Sun et al. [154]	2021	The Comprehensive Oral Health Literacy (COHL)	Chinese general population Community health centers in Beijing(18–86 years)	Oral health literacy	30	✓	EFA; Discriminant, Concurrent	Cronbach α = 0.72	Test-retest = 0.972
Poureslami et al. [155]	2021	Vancouver Airways Health Literacy Tool (VAHLT)	-	Chronic airway disease (CAD) patients	44	✓	-	-	-
Mahmoudian et al. [156]	2021	Hearing health literacy in Iranian young people	Iranian young people (12–25 years)	Hearing health literacy	22	✓	-	Cronbach α = 0.65	-
Simkiss et al. [157]	2021	Knowledge and Attitudes to Mental Health Scales (KAMHS)	Children and adolescents (13–14 years)	Mental health literacy	50	✓	EFA; CFA	Lavaan. Omega(ω) = 0.53–76	Test-retest = 0.40–0.64
Charophasrat et al. [158]	2021	Oral Health Literacy Questionnaire	Thai adults	Oral Health Literacy	21	✓	Known-group; Concurrent	Cronbach α = 0.87	-
Karimi et al. [159]	2021	Sexual health literacy related to HIV/AIDS and sexually transmitted diseases	Iranian young men (19–29 years)	Sexual health literacy	30	✓	-	Cronbach α = 0.79–0.87	ICC = 0.79–0.87
Ma et al. [160]	2021	Reproductive health literacy questionnaire	Chinese unmarried youth (15–24 years)	Reproductive health literacy	58	✓	CFA	Cronbach α = 0.91; split-half reliability = 0.84	Test-retest = 0.72
Suto et al. [161]	2021	Health literacy scale for preconception care	Japanese adults (16–49 years)	Reproductive health literacy	17 & 25	✓	EFA; Criterion	Cronbach α = 0.68–0.89 & 0.82–0.90	-
Kodama et al. [162]	2021	Mental Health Literacy Scale for Depression Affecting the Help-Seeking Process	Health Professional Students	Mental health literacy	10	✓	EFA; CFA; Criterion	Cronbach α = 0.68–0.85	Test-retest (ICC) = 0.78
Aller et al. [163]	2021	Mental Health Awareness and Advocacy Assessment Tool (MHAA-AT)	college attending participants of Amazon’s Mechanical Turk	Mental health literacy	65	✓	EFA; Convergent	Cronbach α = 0.62–0.95	-
Robbins et al. [164]	2021	OSA Functional Health Literacy (SOFHL)	Dwelling black participants, at risk for OSA	Obstructive sleep apnea functional health literacy	18	-	-	Cronbach α = 0.71–0.81	-
Rabin et al. [165]	2021	Mental Health Literacy Assessment-college (MHLA-c)	US college students	Mental health literacy	54	✓	Known groups	KR-20 = 0.74–0.75	-
Moein et al. [166]	2021	Physical activity health literacy in Iranian older adults (PAHLIO) questionnaire	Iranian older adults (60–75 years)	Physical activity health literacy	18	✓	EFA; CFA	Cronbach α = 0.85–0.94	Test-retest (ICC) = 0.89–1

* Unpublished (dissertation).

### Population- specific instruments

A number of health literacy instruments were designed for a specific population- or specific demographic population (n = 22). The grouping was based on age (adolescents, adults/elderly adults, and the elderly) or nationality (Korean, Taiwanese, English, Spanish, American, Switzerland, Australian, German, Chinese, Iranian, and Finnish). A list of instruments and their psychometric properties are shown in Table 5.

**Table 5:** Population- specific health literacy instruments (1993–2021).

Author [ref.]	year	Name (abbreviation)	Country/sample	Items	Validity		Reliability	
					Face/Content	Construct	Internal consistency	External
Lee TW et al. [167]	2009	Korean Health Literacy Scale (KHLS)	Korean older adults	24	✓	EFA; CFA	Cronbach α = 0.89	-
Pan et al. [168]	2010	Taiwan Health Literacy Scale (THLS)	Taiwanese elderly adults	66	-	Concurrent; Discriminant	Cronbach α = 0.98	-
Tsai et al. [169]	2010	Mandarin Health Literacy Scale (MHLS)	Taiwanese adults	50	✓	EFA; CFA; Convergent; Predictive	Cronbach α = 0.95; Spearman–Brown split-half coefficient = 0.95	-
Weidmer et al. [170]	2012	Consumer Assessment of Healthcare Providers and Systems (CAHPS)	English and Spanish adult patients	22	-	CFA	Cronbach α = 0.89	-
Massey et al. [171]	2013	Multidimensional measure of adolescent health literacy	American adolescent	24	✓	EFA	Cronbach α = 0.83	-
Wang et al. [172]	2014	Multidimensional instrument to assess competencies for health	Switzerland resident population	74	✓	EFA; CFA	Cronbach α = 0.72–0.81	-
Harper et al. [173]	2014	Health literacy assessment for young adult college students	American undergraduate student	51	✓	CFA: IRT	-	-
Yuen et al. [174]	2014	Health Literacy of Caregivers Scale- Cancer (HLCS-C)	Australian cancer caregivers	88	✓	-	-	-
Manganello et al. [175]	2015	he Health Literacy Assessment Scale for Adolescents (HAS-A)	American Teen (12–19)	15	✓	EFA; Criterion	Cronbach α = 0.73–77	-
Shen et al. [176]	2015	Chinese resident health literacy scale	Chinese population-based	64	-	CFA; Discriminant	Cronbach α = 0.95; Spearman–Brown split-half coefficient = 0.94	-
Abel et al. [177]	2015	Short survey tool for public health and health promotion research	German-speaking young adults	8	-	EFA; CFA; Discriminant	Cronbach α = 0.64	-
Ghanbari et al. [178]	2016	Health Literacy Measure for Adolescents (HELMA)	Iranian adolescents	44	✓	EFA	Cronbach α = 0.93	Test-retest (ICC) = 0.93
Paakkari et al. [179]	2016	Health Literacy for School-Aged Children (HLSAC)	Finnish school-aged children	10	✓	CFA	Cronbach α = 0.93	Test-retest = 0.83
Yang et al. [180]	2017	The Health Literacy Index for Female Marriage Immigrants (HLI-FMI)	Asian women	12	-	CFA; Discriminant; Concurrent	Cronbach α = 0.74	-
Ernstmann et al. [181]	2017	Health Literacy-sensitive Communication (HL-COM)	German adult patients	9	-	EFA; CFA	Cronbach α = 0.91; Item-total correlation = 0.622–0.762	-
Chang et al. [182]	2017	Instrument Of Health Literacy Competencies (IOHLC)	Chinese-speaking health professionals	49	-	EFA; CFA; Discriminant; Convergent; IRT	Cronbach α = 0.97	-
Eliason et al. [183]	2017	Health literacy among Lesbian, Gay, and Bisexual (LGB)	American adults	10	✓	EFA	Cronbach α = 0.95	Test-retest = 0.91
Hashimoto et al. [184]	2017	health Literacy Scale among Brazilian Mothers (HLSBM)	Brazilian mothers	10	✓	EFA; CFA; Concurrent	Cronbach α = 066–0.89	-
Bradley-Klug et al. [185]	2017	Health Literacy and Resiliency Scale: Youth version (HLRS-Y)	American youth	37	-	EFA; Discriminant	Cronbach α = 0.88–0.94	-
Guo et al. [186]	2018	Chinese eight-item Health Literacy Assessment Tool (c-HLAT-8)	Chinese secondary school students	8	✓	CFA; Convergent	Cronbach α = 0.94; ICC = 0.72	-
Azizi et al. [187]	2019	Health Literacy Scale for Workers (HELSW)	Iranian workers	34	✓	EFA	Cronbach α = 0.90	Test-retest = 0.69 to 0.86; ICC = 0.72 to 0.84
Domanska et al. [188]	2020	Measurement Of Health Literacy Among Adolescents Questionnaire (MOHLAA-Q)	German adolescents	29	✓	Convergent; Concurrent; CFA	Cronbach α = 0.79	-

### e-Health literacy instruments

There were 11 electronic health literacy instruments. Of these, the instrument developed by Norman et al. [189] was used more frequently in various studies. A list of instruments is presented in Table 6.

**Table 6:** Electronic health literacy instruments (1993–2021).

Author [ref.]	year	Name (abbreviation)	Country/sample	Items/Terms/phrases	Validity		Reliability	
					Face/Content	Construct	Internal consistency	External
Norman et al. [189]	2006	The e-Health Literacy Scale (e-HEALS)	Canadian youth	8	✓	EFA	Cronbach α = 0.88	Test-retest = 0.40–0.68
Hahn et al. [190]	2011	Health Literacy assessment using Talking Touchscreen (Health LiTT)	American English speaking patients	82	✓	IRT; Discriminant	Cronbach α≥ 0.9	-
Ownby et al. [191]	2013	Fostering Literacy for Good Health Today (FLIGHT) & Vive Desarollando Amplia Salud (VIDAS)	Spanish and English speaking adults	82	✓	EFA; Concurrent; Know groups	Cronbach α = 0.56–0.83	-
Seçkin et al. [192]	2016	Electronic Health Literacy Scale (e-HLS-19)	American residents adults	19	-	EFA; CFA	Cronbach α = 0.93; Item total correlations = 0.09–0.81	-
Van der Vaart et al. [193]	2017	Digital Health Literacy Instrument (DHLI)	General Dutch population	21	✓	EFA	Cronbach α> 0.68–0.88	ICC = 0.77
Kayser et al. [194]	2018	English/Danish version of e-Health Literacy Questionnaire (eHLQ)	English/Danish people with chronic conditions	35	-	IRT; EFA; CFA	Cronbach α> 0.7	-
Paige et al. [195]	2019	Transactional e-Health Literacy Instrument (TeHLI)	American patients	18	-	CFA	Cronbach α = 0.90	-
Woudstra et al. [196]	2019	Computer-based and performance-based instrument to assess health literacy skills for informed decision making in colorectal cancer screening	Dutch adults	22	-	IRT; CFA; Convergent; Predictive	Cronbach α = 0.66	-
Castellvi et al. [197]	2020	Espaijove.net Mental Health Literacy test (EMHL)	Spanish adolescents	35	-	Groups known; Convergent	Cronbach α = 0.610 & 0.74	Test-retest (ICC) = 0.57 & 0.42
Liu et al. [198]	2021	eHealth Literacy Scale (eHLS-Web 3.0)	Chinese college students	24	✓	Convergent, Concurrent; EFA; CFA	Cronbach α = 0.97	Test-retest = 0.85
Duong et al. [199]	2021	eHealthy Diet Literacy Questionnaire (e-HDLQ)	Taiwanese adults aged 18 years and above	11	✓	EFA; Convergent	Cronbach α = 0.64	-

### Other versions that reported for an original instrument

There were a number of instruments that translated and validated in other nations with different demographic backgrounds (n = 199). A list of these instruments is presented in Table 7.

**Table 7:** The original health literacy instruments and the existing translations and validation versions (1993–2021).

**General health literacy instruments**			
**Author [ref.]**	**Original instrument [abbreviation]**	**Translations**	**Validation and other versions**
Davis et al. [14]	Rapid Estimate of Adult Literacy in Medicine (REALM)	UK [200]; Korean American [201]; Arabic [202];	REALM-SF [203]; REAL-G [204, 205]; REAL-VS [206]; REALM-Teen [207, 208]; REALD-30 [209–211]; REALD-20 [212]; REALD-99 [213]; OHLA [214, 215]
Parker et al. [15]	Test of Functional Health Literacy in Adults (TOFHLA)	Serbian [216]; Danish [217]; American [218]; Albanian [219];	TOFHLA in dentistry (TOFHLiD) [220]; OA‐TOFHLiD [221]
Baker et al. [41]	Short form of the Test of Functional Health Literacy in Adults (S-TOFHLA)	Korean American [201]; Arabic [202, 222, 223]; Serbian [216]; Turkish [224]; Spanish [225]; Chinese [226]; Italian [66]; American [227]; Chines [228]; Hebrew [229]; English-Spanish [230]	-
Weiss et al. [16]	Newest Vital Sign (NVS)	American [208, 227, 231, 232]; Brazilian Portuguese [233, 234]; Italian [66, 235]; Taiwanese [236]; Brazilian [237]; UK [238]; Dutch [239]; Turkish [240]; Arabic [223, 241];	-
Lee et al. [42]	Short Assessment of Health Literacy for Spanish-speaking Adults (SAHLSA-50)	Dutch [242]; Portuguese [243–245]; Dutch [246]; Spanish & English [247]	SAHLPA-33 [248]
Morris et al. [43]	Single Item Literacy Screener (SILS)	Arabic [202, 222, 223]; Italian [66, 249]; American [227]	-
Zikmund-Fisher et al. [44]	Subjective Numeracy Scale (SNS)	English-Spanish [230]; American [250]	-
Ishikawa et al. [45]	Functional, Communicative, and Critical Health Literacy (FCCHL)	German [251]; Dutch [252]; French [253]; Iranian [254]; Japanese [255]; Australian [256]; American [257, 258]; Korean [259]; Swedish [260];	FCCHL-12 [261]
Chew et al. [46]	Health Literacy Screening Questions	English-Spanish [230]; American [262–265]; American-English and Spanish [266]; Hungary/Italy/Lebanon/Switzerland/Turkey [267]	-
Pleasant et al. [47]	Public Health Literacy Knowledge Scale	Turkish [268]	-
Rawson et al. [48]	Medical Term Recognition Test (METER(	Italian [269]; Portuguese [270]	-
McCormack et al. [50]	Health Literacy Skills Instrument	-	HLSI-SF-10 [271]
Osborne et al. [55]	Health Literacy Questionnaire (HLQ)	Danish [272]; Slovak [273]; Norwegian [274]; Ghanaian [275]; German [276]; Australian [277–280]; Chinese [281, 282]; Urdo [283]; Norwegian [284]; Yoruba [285]; Brazilian [286]; Brazilian Portuguese [287]; French [288, 289]; American [290]	-
Sorensen et al. [56]	European Health Literacy Survey Questionnaire (HLS-EU-Q-47)	Albanian [219]; Turkish [291]; Indonesian/Kazakh/ Malay/Myanmar/Burmese/Mandarin/Vietnamese [292]; Taiwanese [293–296]; Norwegian [297]; Japanese [298]; Vietnamese [299]	-
Suka et al. [57]	14-item Health Literacy Scale (HLS-14)	Brazilian Portuguese [300]	-
Pelikan et al. [61]	Short versions of the European Health Literacy Survey Questionnaire (HLS-EU-Q16, Q6)	Turkish [301]; Italian [302]; Icelandic [303]; French [304]; Arabic/French [305]; Swedish-Arabic [306]; Japanese [307]; Brazilian Portuguese [308]; Pakistanian [309]; German [310]; French [311]	-
Haghdoost et al. [65]	Iranian Health Literacy Questionnaire (IHLQ)	Iranian [312]	-
Finbraten et al. [69]	Short version of Health Literacy Survey Questionnaire (HLS-Q12)	Japanese [307]	-
Duong et al. [71]	European Health Literacy Survey Questionnaire (HLS-SF12)	Taiwanese [313]; Vietnam [314]; Turkish [315]; Japanese [307]	-
**Disease specific health literacy instruments**			
Huizinga et al. [77]	Diabetes Numeracy Test (DNT-43, 15)	-	DNT-5 [230]
Kim et al. [78]	High Blood Pressure-focused Health Literacy Scale (HBP-HLS)	Chinese [316]	-
Leung et al. [79]	Chinese Health Literacy Scale for Diabetes (CHLSD)	Chinese [317]	
Dumenci et al. [84]	Cancer Health Literacy Along a Continuum (CHLT-30) & (CHLT-6)	American [318]; Chinese [319]	-
Matsuoka et al. [87]	Heart Failure-specific Health Literacy scale (HF-specific HL)	Chinese [320]; Iranian [321]	-
**Content specific health literacy instruments**			
Cormier et al. [111]	Health Literacy Knowledge and Experience Survey (HL-KES)	Iranian [322]	--
Sabbahi [112]	Oral Health Literacy Instrument (OHLI)	Russian [323]; Chilean [324]; Malaysian [325]	-
Kumar et al. [113]	Health Literacy, Numeracy and The Parental Health Literacy Activities Test (PHLAT)	Spanish [326]	-
Wong et al. [118]	Hong Kong Oral Health Literacy Assessment Task for Pediatric Dentistry (HKOHLAT-P)	Brazilian-Portuguese [327]	-
Jones et al. [120]	Health Literacy in Dentistry scale (HeLD-29)	Thai [328]; Australian [329]; Brazilian [330, 331]	He LD‐14 [332]
Naghibi Sistani et al. [121]	Oral health literacy for Adults Questionnaire (OHL-AQ)	American [333, 334]; Persian [335]; Hindi [336]; Mandarin [337]	-
Shreffler-Grant et al. [123]	Montana State University (MSU) CAM Health Literacy Scale	American [338]	-
O’Connor et al. [125]	Mental Health Literacy Scale (MHLS)	Pakistani [339]; South African and Zambian [340]; Arabic [341]; Chinese [342]; Portuguese [343]; Iranian [344–348];	-
Jung [132]	Multicomponent Mental Health Literacy Measure (MMHLM)	-	MMHLM for Student Athletes and Therapists [349]
Campos et al. [133]	Mental Health Literacy (MHLq)	Portuguese [350]	-
Matsumoto et al. [138]	Social Determinants of Health Questionnaire (HL-SDHQ)	Korean [351]	-
**Population- specific health literacy instruments**			
Lee TW et al. [167]	Korean Health Literacy Scale (KHLS)	Korean [352]	-
Pan et al. [168]	Taiwan Health Literacy Scale (THLS)	-	STHLS [353]; THLS for Middle-Aged and Older People [354]
Tsai et al. [169]	Mandarin Health Literacy Scale (MHLS)	-	S-MHLS [355]
Yuen et al. [174]	Health Literacy of Caregivers Scale- Cancer (HLCS-C)	Australian [356]	-
Manganello et al. [175]	Health Literacy Assessment Scale for Adolescents (HAS-A)	Arabic [357]	-
Paakkari et al. [179]	Health Literacy for School-Aged Children (HLSAC)	Turkish [358]; Polish [359]; Danish [360]; Finnish/Polish/Slovak/Belgian [361]	-
**Electronic health literacy instruments**			
Norman et al. [189]	e-Health Literacy Scale (e-HEALS)	Swedish-Arabic [306]; Italian [362–364]; Portuguese [365]; Dutch [366]; Hungarian [367]; Greek and Cypriot [368]; African-American and Caucasian [369]; US, UK, New Zealand [370]; UK [371]; American-Hispanic [372]; American [373–375]; Taiwanese [199]; Indonesian [376]; Polish [377]; Australian [378]; Korean [379, 380]; Arabic [381]; Iranian [382, 383]; Serbian [384]; Norwegian [385]; Ethiopian [386]; Swiss-German [387]; Brazilian [388, 389]; Chinese [390–392]	-
Hahn et al. [190]	Health Literacy Assessment Using Talking Touchscreen Technology (Health LiTT)	-	10-item Health LiTT [393]
Van der Vaart et al. [193]	Digital Health Literacy Instrument (DHLI)	American [394]	-
Kayser et al. [194]	English/Danish version of e-Health Literacy Questionnaire (eHLQ)	Australian [395]	-

### Results for quality assessment

As indicated in the methods section, all papers under review were assessed for quality. The results are shown in Table 8.

**Table 8:** The results for quality assessment of existing health literacy instruments (1993–2021).

Author [ref.]	Reliability		Validity						Ratings
			Content & face	Construct					
				Structural		Criterion	Hypothesis testing		
	Internal Consistency	Test- retest (ICC)		EFA	CFA	Predictive & Concurrent	Convergent	Discrimination & Known groups comparison	
**General health literacy instruments**									
Davis et al. [14]	✓	✓	**-**	**-**	**-**	✓	**-**	**-**	Fair
Parker et al. [15]	✓	**-**	✓	**-**	**-**	✓	**-**	**-**	Fair
Baker et al. [41]	✓	**-**	**-**	**-**	**-**	✓	**-**	**-**	Fair
Weiss et al. [16]	✓	**-**	**-**	**-**	**-**	✓	**-**	**-**	Fair
Lee et al. [42]	✓	✓	**-**	**-**	✓	✓	✓	**-**	Good
Morris et al. [43]	**-**	**-**	**-**	**-**	**-**	✓	-	**-**	Poor
Zikmund-Fisher et al. [44]	**-**	**-**	**-**	**-**	**-**	✓	-	**-**	Poor
Ishikawa et al. [45]	✓	-	**-**	✓	**-**	-	-	✓	Fair
Chew et al. [46]	-	-	**-**	**-**	**-**	✓	-	-	Poor
Pleasant et al. [47]	✓	-	✓	**-**	**-**	-	-	✓	Fair
Rawson et al. [48]	✓	-	**-**	**-**	**-**	✓	-	-	Fair
Zhang et al. [49]	✓	✓	**-**	**-**	**-**	-	✓	✓	Good
McCormack et al. [50]	✓	**-**	✓	**-**	✓	✓	-	-	Good
Yu Ko et al. [51]	✓	-	✓	-	-	✓	✓	-	Good
Begoray et al. [52]	✓	-	-	-	-	✓	-	-	Fair
Kaphingst et al. [53]	-	✓	-	-	-	✓	-	-	Fair
Helitzer et al. [54]	-	-	✓	-	-	-	✓	-	Fair
Osborne et al. [55]	✓	-	✓	-	✓	-	-	✓	Good
Sorensen et al. [56]	✓	-	✓	✓	-	-	-	-	Fair
Suka et al. [57]	✓	-	-	✓	✓	-	-	-	Fair
Farin et al. [58]	✓	-	✓	✓	✓	-	-	-	Good
Jordan et al. [59]	✓	✓	✓	✓	✓	-	-	-	Good
Sand-Jecklin [60]	✓	-	**-**	✓	**-**	✓	-	-	Fair
Pelikan et al. [61]*	✓	-	✓	-	✓	✓	-	-	Good
Kang et al. [62]	✓	✓	✓	✓	✓	-	-	-	Good
Nakagami et al. [63]	✓	-	✓	-	-	✓	✓	-	Good
Chau et al. [64]	✓	✓	✓	✓	✓	✓	-	✓	Excellent
Haghdoost et al. [65]	✓	✓	✓	✓	-	-	-	**-**	Good
Zotti et al. [66]	-	-	✓	-	-	-	✓	✓	Fair
Tsubakita et al. [67]	✓	-	-	✓	-	✓	-	-	Fair
Kim [68]	✓	-	-	-	-	-	✓	-	Fair
Finbraten et al. [69]	✓	-	-	-	✓	-	✓	-	Fair
Pleasant et al. [70]	✓	-	-	-	-	-	-	✓	Fair
Duong et al. [71]	✓	-	-	-	✓	-	✓	-	Fair
Mc Clintock et al. [72]	✓	-	✓	✓	-	-	-	✓	Good
Leung et al. [73]	-	-	-	-	-	✓	-	-	Poor
Shannon et al. [74]	-	✓	✓	-	-	-	-	-	Fair
Tavousi et al. [75]	✓	-	✓	✓	-	-	-	-	Fair
Park et al. [76]	✓	-	✓	✓	✓	✓	-	-	Good
**Disease specific health literacy instruments**									
Huizinga et al. [77]	✓	-	✓	✓	-	-	✓	✓	Good
Kim et al. [78]	✓	-	✓	-	-	-	✓	✓	Good
Leung et al. [79]	✓	✓	✓	-	✓	-	-	✓	Good
Leung et al. [80]	✓	✓	✓	-	-	-	-	✓	Good
Ownby et al. [81]	✓	-	-	✓	-	✓	✓	-	Good
Sun et al. [82]	✓	-	-	✓	✓	-	-	-	Fair
Han et al. [83]	✓	-	✓	-	-	✓	✓	✓	Good
Dumenci et al. [84]	✓	✓	✓	-	✓	-	-	✓	Good
Londono et al. [85]	-	✓	✓	-	-	-	-	-	Fair
Shih et al. [86]	✓	-	✓	-	✓	-	-	-	Fair
Matsuoka et al. [87]	✓	✓	✓	✓	-	-	-	✓	Good
Tian et al. [88]	✓	-	✓	✓	-	-	-	✓	Good
Mafutha et al. [89]	-	-	✓	-	-	✓	-	-	Fair
Tique et al. [90]	✓	-	✓	✓	-	-	✓	-	Good
Chou et al. [91]	✓	-	✓	-	✓	✓	-	-	Good
Yang et al. [92]	✓	-	-	-	✓	-	-	✓	Fair
Lee et al. [93]	✓	✓	✓	✓	✓	✓	✓	-	Excellent
Khazaei et al. [94]	✓	✓	✓	✓	✓	-	-	-	Good
Dehghani et al. [95]	✓	✓	✓	✓	-	-	-	✓	Good
Yeh et al. [96]	✓	-	✓	-	✓	-	-	-	Fair
Kang et al. [97]	✓	✓	✓	✓	✓	✓	-	-	Excellent
Tutu et al. [98]	✓	-	✓	✓	-	-	-	-	Fair
Cardoso et al. [99]	✓	✓	✓	-	-	-	-	-	Fair
De Sousa et al. [100]	✓	✓	✓	-	-	✓	-	-	Good
Li et al. [101]	✓	✓	✓	✓	✓	-	-	✓	Excellent
Wu et al. [102]	✓	✓	✓	-	✓	-	-	-	Good
Martins et al. [103]	-	✓	✓	-	-	-	-	-	Fair
Echeverri et al. [104]	✓	-	✓	✓	✓	-	-	✓	Good
Huang et al. [105]	✓	-		-	-	-	-	-	Poor
Rajabi et al. [106]	✓	-	✓	✓	-	-	-	-	Fair
Wei et al. [107]	✓	-	✓	-	✓	-	-	-	Fair
Chen et al. [108]	✓	✓	✓	✓	✓	-	-	-	Good
Savci et al. [109]	✓	-	✓	✓	✓	✓	-	-	Good
Hiltrop et al. [110]	✓	-	-	✓	✓	-	✓	-	Good
**Content specific health literacy instruments**									
Cormier et al. [111]*	✓	-	✓	✓	-	-	-	-	Fair
Sabbahi et al. [112]	✓	✓	✓	-	-	✓	✓	✓	Excellent
Kumar et al. [113]	✓	-	✓	-	-	-	-	✓	Fair
Macek et al. [114]	✓	-	✓	-	-	✓	-	-	Fair
Devi et al. [115]	✓	✓	-	-	-	✓	✓	-	Good
Mojoyinola [116]	✓	-	-	-	-	-	-	-	Poor
Loureiro et al. [117]	✓	-	-	✓	-	-	-	-	Fair
Wong et al. [118]	✓	✓	✓	-	-	✓	✓	-	Good
Dahlke et al. [119]	-	-	✓	-	-	✓	✓	-	Fair
Jones et al. [120]	✓	✓	✓	✓	-	✓	✓	✓	Excellent
Naghibi Sistani et al. [121]	✓	✓	✓	-	-	-	-	✓	Good
Paez et al. [122]	✓	-	-	✓	✓	-	✓	-	Good
Shreffler-Grant et al. [123]	✓	-	✓	✓	-	-	✓	-	Good
Villanueva Vilchis et al. [124]	✓	✓	✓	-	-	-	✓	-	Good
O’Connor et al. [125]	✓	✓	✓	✓	-	✓	-	✓	Excellent
Altin et al. [126]	✓	-	-	✓	✓	✓	-	-	Good
Curtis et al. [127]	✓	-	-	-	✓	✓	✓	-	Good
Guttersrud et al. [128]	✓	-	-	-	-	-	-	-	Poor
Stein et al. [129]	✓	✓	✓	-	-	✓	-	-	Good
Intarakamhanga et al. [130]	✓	-	✓	✓	✓	-	-	-	Good
Kapoor et al. [131]	✓	✓	✓	-	-	✓	✓	-	Good
Jung et al. [132]	✓	-	✓	✓	✓	-	✓	✓	Excellent
Campos et al. [133]	✓	✓	✓	✓	-	-	-	-	Good
Squires et al. [134]	✓	-	✓	✓	-	-	-	-	Fair
Bjornsen et al. [135]	✓	✓	✓	✓	✓	-	-	✓	Excellent
Moll et al. [136]	✓	-	-	✓	-	-	✓	✓	Good
Intarakamhang et al. [137]	✓	-	-	✓	✓	-	-	-	Fair
Matsumoto et al. [138]	✓	-	✓	-	✓	-	-	-	Fair
Tsaia et al. [139]	✓	-	-	✓	✓	✓	✓	-	Good
Lichtveld et al. [140]	✓	-	✓	✓	✓	-	-	-	Good
Areerak et al. [141]	✓	✓	✓	✓	✓	-	-	✓	Excellent
Zhang et al. [142]	✓	✓	✓	-	✓	-	-	-	Good
Irvin et al. [143]	✓	-	✓	✓	-	✓	-	✓	Good
Wei et al. [144]	✓	-	✓	✓	-	-	-	✓	Good
Ayre et al. [145]	✓	-	✓	-	✓	✓	-	-	Good
Intarakamhang et al. [146]	✓	-	✓	-	✓	-	-	-	Fair
Suthakorn et al. [147]	✓	-	✓	✓	✓	-	-	-	Good
Lin et al. [148]	✓	-	✓	✓	-	-	✓	✓	Good
Taheri et al. [149]	✓	✓	✓	✓	-	-	-	-	Good
Tabacchi et al. [150]	✓	-	✓	-	✓	-	-	✓	Good
Zenasa et al. [151]	✓	-	✓	✓	✓	-	-	-	Good
Taoufik et al. [152]	✓	✓	✓	-	-	-	✓	-	Good
Chao et al. [153]	✓	-	✓	✓	✓	-	✓	✓	Excellent
Sun et al. [154]	✓	✓	✓	✓	-	✓	-	✓	Excellent
Poureslami et al. [155]	-	-	✓	-	-	-	-	-	Poor
Mahmoudian et al. [156]	✓	-	✓	-	-	-	-	-	Fair
Simkiss et al. [157]	✓	✓	✓	✓	✓	-	-	-	Good
Charophasrat et al. [158]	✓	-	✓	-	-	✓		✓	Good
Karimi et al. [159]	✓	✓	✓	-	-	-	-	-	Fair
Ma et al. [160]	✓	✓	✓	-	✓	-	-	-	Good
Suto et al. [161]	✓	-	✓	✓	-	✓	-	-	Good
Kodama et al. [162]	✓	✓	✓	✓	✓	✓	-	-	Excellent
Aller et al. [163]	✓	-	✓	✓	-	-	✓	-	Good
Robbins et al. [164]	✓	-	-	-	-	-	-	-	Poor
Rabin et al. [165]	✓	-	✓	-	-	-	-	✓	Fair
Moein et al. [166]	✓	✓	✓	✓	✓	-	-	-	Good
**Population-specific health literacy instruments**									
Lee TW et al. [167]	✓	-	✓	✓	✓	-	-	-	Good
Pan et al. [168]	✓	-	-	-	-	✓	-	✓	Fair
Tsai et al. [169]	✓	-	✓	✓	✓	✓	✓	-	Excellent
Weidmer et al. [170]	✓	-	-	-	✓	-	-	-	Fair
Massey et al. [171]	✓	-	✓	✓	-	-	-	-	Fair
Wang et al. [172]	✓	-	✓	✓	✓	-	-	-	Good
Harper et al. [173]	-	-	✓	-	✓	-	-	-	Fair
Yuen et al. [174]	-	-	✓	-	-	-	-	-	Poor
Manganello et al. [175]	✓	-	✓	✓	-	✓	-	-	Good
Shen et al. [176]	✓	-	-	-	✓	-	-	✓	Fair
Abel et al. [177]	✓	-	-	✓	✓	-	-	✓	Good
Ghanbari et al. [178]	✓	✓	✓	✓	-	-	-	-	Good
Paakkari et al. [179]	✓	✓	✓	-	✓	-	-	-	Good
Yang et al. [180]	✓	-	-	-	✓	✓	-	✓	Good
Ernstmann et al. [181]	✓	-	-	✓	✓	-	-	-	Fair
Chang et al. [182]	✓	-	-	✓	✓	-	✓	✓	Good
Eliason et al. [183]	✓	✓	✓	✓	-	-	-	-	Good
Hashimoto et al. [184]	✓	-	✓	✓	✓	✓	-	-	Good
Bradley-Klug et al. [185]	✓	-	-	✓	-	-	-	✓	Fair
Guo et al. [186]	✓	-	✓	-	✓	-	✓	-	Good
Azizi et al. [187]	✓	✓	✓	✓	-	-	-	-	Good
Domanska et al. [188]	✓	-	✓	-	✓	✓	✓	-	Good
**Electronic health literacy instruments**									
Norman et al. [189]	✓	✓	✓	✓	-	-	-	-	Good
Hahn et al. [190]	✓	-	✓	-	-	-	-	✓	Fair
Ownby et al. [191]	✓	-	✓	✓	-	✓	-	✓	Good
Seçkin et al. [192]	✓	-	-	✓	✓	-	-	-	Fair
Van der Vaart et al. [193]	✓	✓	✓	✓	-	-	-	-	Good
Kayser et al. [194]	✓	-	-	✓	✓	-	-	-	Fair
Paige et al. [195]	✓	-	-	-	✓	-	-	-	Fair
Woudstra et al. [196]	✓	-	-	-	✓	✓	✓	-	Good
Castellvi et al. [197]	✓	✓	-	-	-	-	✓	✓	Good
Liu et al. [198]	✓	✓	✓	✓	✓	✓	✓	-	Excellent
Duong et al. [199]	✓	-	✓	✓	-	-	✓	-	Good

### Synthesis of findings

Numerous instruments have been developed during the past thirty years for measuring health literacy. This review could provide information on 162 instruments. Of these, there were two well-developed instruments:

1. HLQ, which avoided the use of prevailing theories until the later development process, and great care was taken to fully understand the experiences and lives of people, professionals, and healthcare providers [55].
2. HLS-EU-Q47, which used conceptual-based, multi-faceted attributes [56].

However, they reported limited psychometric properties. Of the remaining instruments, 15 instruments reported proper psychometric properties needed. In addition, there were a number of instruments that were translated and validated to other languages more frequently. A list of instruments is presented in Table 9.

**Table 9:** A list of instruments that well developed, reported proper psychometric properties, and instruments frequently translated or validated in other countries (1993–2021).

	Instruments
**Well-developed instruments**	
	Health Literacy Questionnaire (HLQ) (validity-driven) [55]
	Health Literacy Survey Questionnaire (HLS-EU-Q-47) (conceptual-based, multi-faceted attributes) [56]
**Instruments with excellent reported psychometric properties**	
	Chinese Health Literacy Scale for Low Salt Consumption-Hong Kong population (CHLSalt-HK) [64]
	Comprehensive Diabetes Health Literacy Scale (DHLS) [93]
	Korean Health Literacy Scale for Diabetes Mellitus (KHLS-DM) [97]
	Chinese Health Literacy Scale for Tuberculosis (CHLS-TB) [101]
	Oral Health Literacy Instrument (OHLI) [112]
	Health Literacy in Dentistry scale (HeLD-29) [120]
	Mental Health Literacy Scale (MHLS) [125]
	Multicomponent mental health literacy measure [132]
	Mental Health-Promoting Knowledge (MHPK-10) [135]
	Neck pain-specific Health Behavior in Office Workers (NHBOW) [141]
	Mental Health Literacy Scale for Healthcare Students (MHLS-HS) [153]
	The Comprehensive Oral Health Literacy (COHL) [154]
	Mental Health Literacy Scale for Depression Affecting the Help-Seeking Process [162]
	Mandarin Health Literacy Scale (MHLS) [169]
	eHealth Literacy Scale (eHLS-Web 3.0) [198]
**Frequently translated or validated (more than ten)**	
	Rapid Estimate of Adult Literacy in Medicine (REALM) [14]
	Short form of the Test of Functional Health Literacy in Adults (S-TOFHLA) [41]
	Newest Vital Sign (NVS) [16]
	Functional, Communicative, and Critical Health Literacy (FCCHL) [45]
	Health Literacy Questionnaire (HLQ) [55]
	European Health Literacy Survey Questionnaire (HLS-EU-Q-47) [56]
	Short versions of the European Health Literacy Survey Questionnaire (HLS-EU-Q16, Q6) [61]
	e-Health Literacy Scale (e-HEALS) [189]

## Discussion

This bibliometric review covered the literature for about thirty years. The present review extracted and reported a wide range of health literacy instruments in several sections and perhaps could be a good reference for investigators who wish to use an instrument for measuring health literacy. In addition, the current study might help to avoid adding yet another measure to a rather long list of existing instruments.

Some general health literacy instruments have multiple versions used in different languages and populations. For instance, there were 16 versions for the REALM [14], 15 versions for the NVS [16], 6 versions for the TOFHLA [15], 13 versions for the S- TOFHLA [41], and 19 versions for the HLQ [55] (Table 7). Among the general health literacy instruments the HLS-EU-Q [56], which examines health literacy in three areas (health care, health prevention, and health promotion), has a potential to be used universally.

Despite a large number of general health literacy assessment instruments and specific topics, currently having a unique and international instrument for measuring health literacy is one of the concerns of public health professionals. This study showed that one of the most widely used instruments at the international level is the European Health Literacy Survey (HLS-EU-Q) [56]. During the development process, the English version of the HLS-EU-Q simultaneously was translated into Bulgarian, Dutch, German, Greek, Polish, Spanish, Irish, Austrian [56] and in Asia into Indonesia, Kazakhstan, Malaysia, Myanmar, Taiwan, and Vietnam [292]. Also, the Taiwanese [293–296]; Norwegian [297]; Japanese [298]; Vietnamese [299] versions of this instrument have been used in various populations, making it one of the most widely used internationally. Given this instrument’s relatively wide range of applications, it may be considered a prelude for producing an international instrument for measuring health literacy.

Many instruments were developed to measure health literacy among specific diseases (chronic non-communicable diseases, especially diabetes, hypertension, and cancer). With the widespread prevalence of chronic non-communicable, there was a strong desire to develop such instruments. As shown in Table 3, among chronic diseases, diabetes has received more attention than other diseases. Among the instruments that consider a specific content (e.g., maternal, parental, environmental, obesity, and weight gain), oral/dental health literacy and mental health literacy have received special attention.

Development and psychometric evaluation of health literacy instruments was observed in different countries. We recognized health literacy instruments in different languages such as Korean, Taiwanese, English, Spanish, American, Australian, German, Switzerland, Finnish, Iranian, Chinese, Japanese, Brazilian, Philippines, and Vietnamese. As shown in Table 5, the countries of Southeast Asia, especially China, have a long history of activity in this field. It has also been shown that the American population and the populations of Southeast Asian countries (Chinese, Taiwanese, and Koreans) address a large number of health literacy assessment instruments.

One of the unique features of this study is the reporting of e-health literacy instruments. There were eleven instruments available for measuring e-health literacy (Table 6). The existence of many different versions of such instruments (Table 7) demonstrates a growing tendency to measure health literacy related to the increasing use of interment and social media by the general public almost everywhere.

Finally, one should note that the most important question is, do we need so many instruments for measuring health literacy? Although one could not prevent investigators from developing new instruments, it is evident that such haphazard development of instruments is not helpful. It seems that we need a core global general health literacy instrument for use around the globe. Then perhaps it is possible to add a few contents/disease-specific, population- specific, or e-health literacy items to the general instruments according to their use. The experience of the European Organization for Research and Treatment of Cancer-EORTC (the Quality of Life Study Group) might be useful to be adapted (https://qol.eortc.org/quality-of-life-group/).

### Limitations

The main criterion in extracting information was the availability of the full-text papers. In cases of no access to the original text, the required information was extracted from their abstracts. Otherwise, such studies were removed from the review. In addition, we only reviewed papers that included the word health literacy in the title. Thus there is a risk of missing papers that did not use health literacy in their titles.

## Conclusion

This review highlighted that there were more than enough instruments for measuring health literacy. In addition, we found that a number of instruments did not report psychometric properties sufficiently. However, evidence suggest that well developed instruments and those reported adequate measures of validation could be helpful if appropriately selected based on objectives of a given study. Perhaps an authorized institution such as World Health Organization should take responsibility and provide a clear guideline for measuring health literacy as appropriate.

## Supporting information

S1 Checklist PRISMA 2020 checklist. (DOCX)
