# Supplementary material for: Measuring health literacy: A systematic review and bibliometric analysis of instruments from 1993 to 2021
Source: PLoS One. 2022 Jul 15;17(7):e0271524. doi: 10.1371/journal.pone.0271524 (PMC9286266; doi:10.1371/journal.pone.0271524)
Supplement: Attachment — Submitted filename: Response to Editor.docx [file pone.0271524.s002.docx]

**Dr. Lucinda Shen,**

Academic Editor

PLOS ONE

14 February 2022

**Dear Dr. Shen,**

A systematic review and bibliometric analysis of instruments from 1993 to 2021

PLOS ONE

Thank you for your e-mail. This is a re-submission of the above manuscript (PONE-D-21-39359). As requested we have up-dated the review and all changed were made. Please find the following point-by-point responses to earlier editorial comments. Hope you find revisions satisfactory.

Wish you all the best.

Yours sincerely

Ali Montazeri

**Editor Comments:**

In the case of your submission, we are concerned that the literature review was completed in 2021. Systematic and scoping reviews should provide an up-to-date overview of the research topic covered. Since the literature search was completed over a year ago, we are concerned that this study does not meet this requirement.

Thank you. Done.

Furthermore, if you choose to resubmit, please provide a table reporting in detail the results of your quality assessment, showing how each included study scored on every item of the COSMIN Risk of Bias checklist.

Thank you. Done.
