# Supplementary material for: Measuring health literacy: A systematic review and bibliometric analysis of instruments from 1993 to 2021
Source: PLoS One. 2022 Jul 15;17(7):e0271524. doi: 10.1371/journal.pone.0271524 (PMC9286266; doi:10.1371/journal.pone.0271524)
Supplement: Attachment — Submitted filename: Response to Reviewers. R2.docx [file pone.0271524.s003.docx]

Carlos Miguel Rios-González

Academic Editor

PLOS ONE

13 May 2022

**Dear Dr.** **Rios-González,**

PONE-D-22-04494

Measuring health literacy: A systematic review and bibliometric analysis of instruments from 1993 to 2021

PLOS ONE

Thank you for your e-mail. We are grateful to reviewers and found their comments very helpful. We have revised the manuscript and now very pleased to submit the revised version for your consideration. All changed are marked in blue and hope you find it satisfactory.

Wish you all the best.

Yours sincerely

Ali Montazeri

**When submitting your revision, we need you to address these additional requirements.**

1. Please ensure that your manuscript meets PLOS ONE's style requirements, including those for file naming. The PLOS ONE style templates can be found at

<https://journals.plos.org/plosone/s/file?id=wjVg/PLOSOne_formatting_sample_main_body.pdf> and

<https://journals.plos.org/plosone/s/file?id=ba62/PLOSOne_formatting_sample_title_authors_affiliations.pdf>

Thank you. Done.

2. We note that you have indicated that data from this study are available upon request. PLOS only allows data to be available upon request if there are legal or ethical restrictions on sharing data publicly. For more information on unacceptable data access restrictions, please see <http://journals.plos.org/plosone/s/data-availability#loc-unacceptable-data-access-restrictions>.

In your revised cover letter, please address the following prompts:

a) If there are ethical or legal restrictions on sharing a de-identified data set, please explain them in detail (e.g., data contain potentially sensitive information, data are owned by a third-party organization, etc.) and who has imposed them (e.g., an ethics committee). Please also provide contact information for a data access committee, ethics committee, or other institutional body to which data requests may be sent.

b) If there are no restrictions, please upload the minimal anonymized data set necessary to replicate your study findings as either Supporting Information files or to a stable, public repository and provide us with the relevant URLs, DOIs, or accession numbers. For a list of acceptable repositories, please see http://journals.plos.org/plosone/s/data-availability#loc-recommended-repositories.

We will update your Data Availability statement on your behalf to reflect the information you provide.

Data availability was revised and now reads as follows:

**Data Availability**

The authors confirm that all data underlying the findings are fully available without restriction. All relevant data are within the paper.

**Review Comments to the Author**

**Reviewer #1**

Professor Montazeri’s and colleague’s study appears to do something quite unusual: reviewing the literature on measurement instrument construction for the or concept of health literacy. His own sentence differs slightly: “This study aimed to review all existing instruments to summarize the current knowledge on the topic.” This reads to me as if either the development of measurement instruments was the subject of the review, or the instruments themselves or rather their interrelationship with outcomes, the precise nature of which would remain in the dark.

Thank you for your comment. The sentence was revised.

This study aimed to review all existing instruments to summarize the current knowledge on the development of existing measurement instruments and their possible translation and validation in other languages different from the original languages.

The results and conclusions formulate rather unspectacular assertions, such as there are enough suitable measures of health literacy, there is a strong tradition of disease-specific, group-specific and technology-specific instruments, a minor problem is that of choosing the right measure. The authors forward the idea that the WHO might set some standards for that.

The question arises: Do we need a costly systematic review for results and conclusions like those mentioned. And if we go back to the original ideas behind systematic reviewing, one of the purposes of systematic reviewing is to come to a decision if there is evidence, say, for the efficacy of treatment A as well as for treatment B. If review studies of quality exist on the issue, they will tell you which is better, A or B. Nothing of this urgency do we find in the study to be reviewed. And moreover, the authors do not review all of the evidence, deliberately. They include only publications about the development of a measure, or its transfer to another country, Study presentation, explanation, structure and language are fine, though.

Thank you for your thoughtful comment. It was useful, and it has led us to think more carefully in summarizing the evidence. Indeed, we now fully revised the following section and added a table for more clarity. Also, we rewrote the previous synthesis of findings and moved it with some corrections to the end of the discussion. We also revised the conclusion to reflect the critics by the respected reviewer:

**A: synthesis of findings**

Numerous instruments have been developed during the past thirty years for measuring health literacy. This review could provide information on 162 instruments. Of these, there were two well-developed instruments:

1. HLQ, which avoided the use of prevailing theories until the later development process, and great care was taken to fully understand the experiences and lives of people, professionals, and healthcare providers [55].

2. HLS-EU-Q47, which used conceptual-based, multi-faceted attributes [56].

However, they reported limited psychometric properties. Of the remaining instruments, 15 instruments reported proper psychometric properties needed. In addition, there were a number of instruments that were translated and validated to other languages more frequently. A list of instruments is presented in Table 9.

**B: Discussion**

Finally, one should note that the most important question is, do we need so many instruments for measuring health literacy? Although one could not prevent investigators from developing new instruments, it is evident that such haphazard development of instruments is not helpful. It seems that we need a core global general health literacy instrument for use around the globe. Then perhaps it is possible to add a few contents/disease-specific, population-specific, or e-health literacy items to the general instruments according to their use. The experience of the European Organization for Research and Treatment of Cancer-EORTC (the Quality of Life Study Group) might be useful to be adapted (<https://qol.eortc.org/quality-of-life-group/>).

**C: Conclusion**

This review highlighted that there were more than enough instruments for measuring health literacy. In addition, we found that a number of instruments did not report psychometric properties sufficiently. However, evidence suggest that well developed instruments and those reported adequate measures of validation could be helpful if appropriately selected based on objectives of a given study. Perhaps an authorized institution such as World Health Organization should take responsibility and provide a clear guideline for measuring health literacy as appropriate.
